# 5,7-Dihydroxyflavone Enhances the Apoptosis-Inducing Potential of TRAIL in Human Tumor Cells via Regulation of Apoptosis-Related Proteins

**DOI:** 10.1155/2013/434709

**Published:** 2013-02-28

**Authors:** Zhenzhen Zhang, Tingmei Ye, Xueting Cai, Jie Yang, Wuguang Lu, Chunping Hu, Zhigang Wang, Xiaoning Wang, Peng Cao

**Affiliations:** ^1^Laboratory of Cellular and Molecular Biology, Jiangsu Province Institute of Traditional Chinese Medicine, 100 Shizi Street, Hongshang Road, Nanjing, Jiangsu 210028, China; ^2^Department of Biology, School of Chemistry and Life Sciences, Lishui University, Lishui, Zhejinag 323000, China

## Abstract

Tumor necrosis factor-related apoptosis-inducing ligand (TRAIL) is a promising candidate for the treatment of cancer, because it preferentially induces apoptosis in numerous cancer cells with little or no effect on normal cells. 5,7-Dihydroxyflavone is a dietary flavonoid commonly found in many plants. Here we show that the combined treatment with 5,7-dihydroxyflavone and TRAIL at subtoxic concentrations induced strong apoptotic response in human hepatocarcinoma HepG2 cells, acute leukemia Jurkat T cells, and cervical carcinoma HeLa cells. We further investigated the mechanisms by which 5,7-dihydroxyflavone augments TRAIL-induced apoptosis in HepG2 cells. 5,7-Dihydroxyflavone up-regulated the expression of pro-apoptotic protein Bax, attenuated the expression of anti-apoptotic proteins Bcl-2, Mcl-1, and IAPs, and reduced the phosphorylation levels of Akt and STAT3, weakening the anti-apoptotic signals thus facilitating the process of apoptosis. Moreover, 5,7-dihydroxyflavone and TRAIL were well tolerated in mice, and the combination of 5,7-dihydroxyflavone and TRAIL reduced tumor burden *in vivo* in a HepG2 tumor xenograft model. Interestingly, 5,7-dihydroxyflavone-mediated sensitization to TRAIL-induced cell death was not observed in normal human hepatocytes L-O2. These results suggest that the 5,7-dihydroxyflavone in combination with TRAIL might be used for cancer prevention and/or therapy.

## 1. Introduction

Tumor necrosis factor-related apoptosis-inducing ligand (TRAIL) is a member of the TNF superfamily that selectively induces apoptosis of a variety of tumor cells and transformed cells, but not most normal cells [1–3]. Therefore, TRAIL has garnered intense interest as a potential effective antitumour therapeutic agent. 

Binding of TRAIL to its death receptor (DR4 and/or DR5) results in trimerization of the receptor, formation of the death-inducing signaling complex (DISC), and subsequently activation of caspase-8 and caspase-10 [3]. Activate caspase-8 and caspase-10 then cleave caspase-3, which in turn cleaves its substrates and eventually executes apoptosis [3]. In type II cells, TRAIL-initiated apoptotic signaling requires an amplification loop through the mitochondrial pathway, in which apoptosis proceeds via release of cytochrome *c* and Apaf-1, resulting in caspase-9 and then caspase-3 activation [4]. 

However, the potential application of TRAIL in cancer therapy is limited, as many human tumors, especially some highly malignant tumors, are partially or completely resistant to the apoptotic effects induced by TRAIL [5–7]. Therefore, combination TRAIL with other agents to overcome the low sensitivity and resistance of cancer cells to TRAIL has been a promising strategy to potentiate the therapeutic applications of TRAIL [8]. 

**Figure 1:**
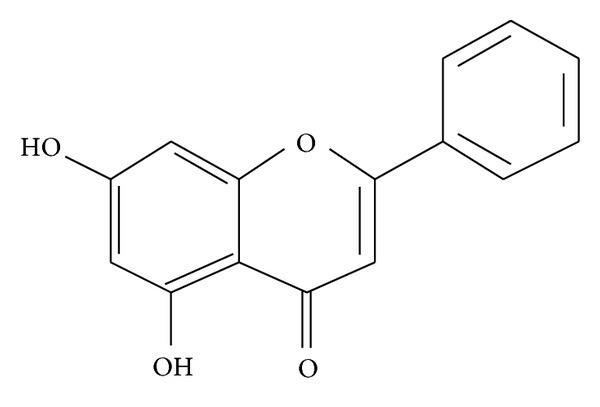
Chemical structure of 5,7-dihydroxyflavone (C_15_H_10_O_4_, CAS No: 480-40-0, Mol. Wt.: 254.24).

5,7-Dihydroxyflavone (Figure 1), a dietary flavonoid, is widely distributed in many plants with high concentrations in honey and propolis [9–11]. Previously, 5,7-dihydroxyflavone has been shown to have strong anti-inflammatory [12], antioxidant [13], and antiviral [14] and anticancer [15, 16] activities. In the current report, we show that 5,7-dihydroxyflavone sensitizes some cancer cell lines to TRAIL-mediated apoptosis while having no effect on normal human hepatocytes L-O2. Our results indicated that 5,7-dihydroxyflavone increased the expression of Bax and decreased the expression of Bcl-2, Mcl-1, and inhibitor of apoptosis proteins (IAPs) in HepG2 cells. Treatment with 5,7-dihydroxyflavone also inhibited the activation of Akt and STAT3. Furthermore, 5,7-dihydroxyflavone acted synergistically with TRAIL to reduce tumor burden in a hepatocarcinoma xenograft model.

## 2. Materials and Methods

### 2.1. Reagents and Antibodies

5,7-Dihydroxyflavone was purchased from Nanjing TCM Institute of Chinese Materia Medica, China. Recombinant human TRAIL was purchased from R&D systems (Minneapolis, MN, USA). (4, 5-Dimethylthiazol-2-yl)-2, 5-diphenyltetrazolium bromide (MTT), Ribonuclease A (RNase A), proteinase K, and propidium iodide (PI) were purchased from Sigma-Aldrich (St. Louis, MO, USA). Hoechst 33258 were purchased from KeyGEN Biotechnology (Nanjing, China). Antibodies against caspase-9, caspase-3, PARP, c-FILP, Bcl-2, Bax, Bcl-X_*L*_, Mcl-1, c-IAP1, c-IAP-2, XIAP, Survivin, p-STAT3 (Ser727), STAT3, and GAPDH were purchased from Cell Signalling Technology (Beverly, MA, USA). Anti-DR4, anti-DR5, mouse IgG1 isotype control, and antimouse IgG-FITC antibodies were from eBioscience (San Diego, CA, USA). Antibodies for p-Akt (Ser473), Akt, p-JNK1/2/3 (Thr183+Tyr185), JNK1/2/3, p-p38 (Tyr182), and p38 were from Bioworld (Minneapolis, MN, USA). Antibodies against p-ERK-1/2 (Thr202/Tyr204), ERK-1/2, and horseradish peroxidase-conjugated secondary antibodies (goat-antirabbit and goat-antimouse) were obtained from Santa Cruz Biotechnology (Santa Cruz, CA, USA). 

### 2.2. Cell Culture

The human hepatocarcinoma HepG2 cells, obtained from the Cell Bank of Type Culture Collection of Chinese Academy of Sciences, and human acute leukemia Jurkat T cells (clone E6-1), obtained from the American Type Culture Collection, were maintained in RPMI-1640 medium (Invitrogen, Carlsbad, CA, USA) supplemented with 10% fetal bovine serum (FBS; Invitrogen, Carlsbad, CA, USA). The human cervical carcinoma HeLa cells and normal human hepatocytes L-O2, obtained from the Cell Bank of Type Culture Collection of Chinese Academy of Sciences, were maintained in DMEM medium (Invitrogen, Carlsbad, CA, USA) supplemented with 10% fetal bovine serum (FBS; Invitrogen, Carlsbad, CA, USA). All cells were cultured in a humidified atmosphere with a 5% CO_2_ incubator at 37°C. 

### 2.3. Cell Viability Assay

Cell viability was assessed using MTT assay. The cells (1 × 10^4^ for HepG2 cells and L-O2 cells, 5 × 10^4^ for Jurkat cells, 7 × 10^3^ for HeLa cells) were seeded in 96-well flat-bottomed microtiter plates in triplicate cultures. After an overnight incubation, cells were treated with 5,7-dihydroxyflavone or TRAIL alone or in combination for the indicated time periods. MTT was prepared at 5 mg/mL in phosphate-buffered saline (PBS) and added to each well. The cell cultures were continued for another 4 h at 37°C. DMSO was added to each well, and the absorbance was measured at 570-nm and 630-nm wavelengths using a microculture plate reader. The cell viability was expressed as a percentage of absorbance in cells with indicated treatments to that in cells with solvent control treatment.

### 2.4. Detection of Morphological Apoptosis

The cells were seeded in 24-well culture dishes overnight prior to treatment in 5,7-dihydroxyflavone or TRAIL alone or in combination for 24 h. Apoptotic nuclear morphology was assessed using Hoechst 33258 staining. The cells were washed twice with PBS and fixed with 4% formaldehyde for 30 min at 4°C. The fixing solution was removed and the cells were washed twice with PBS before staining with Hoechst 33258. After staining for 10 min at room temperature, the cells were washed again and observed under a fluorescence microscope (Zeiss Axio Observer A1) at 340 nm. 

### 2.5. DNA Content Assay

After treated as indicated, the cells were harvested and washed twice in cold PBS. Cell pellets were fixed in 70% ethanol and washed in cold PBS. Then the pellets were suspended in 1 mL of PI solution containing 50 *μ*g/mL of PI, 1 mg/mL RNase A, and 0.1% (w/v) Triton X-100 in 3.8 mM sodium citrate, followed by incubation on ice in the dark for 30 min. Samples were analyzed by FACScan laser flow cytometer (FACSCalibur, Becton Dickinson, USA).

### 2.6. Western Blot Analysis

The cells were treated as indicated and lysed for 5 min at 4°C with ice cold RIPA buffer (1% NP-40 in 150 mmol/L NaCl, 50 mmol/L Tris (pH 7.5), and 2 mmol/L EDTA). The equalized amounts of proteins from each sample were subjected to SDS-polyacrylamide gel electrophoresis. Protein bands were then transferred to polyvinylidene difluoride membranes. Membranes were blocked in TBST (TBS with 0.05% Tween-20) containing 1% (w/v) bovine serum albumin (BSA), washed in TBST, and then incubated with primary antibody. After washing, membranes were incubated with secondary antibody conjugated with IgG horseradish peroxidase (HRP). Immune complexes were detected by the enhanced chemiluminescence system. GAPDH was used as loading control. 

### 2.7. Flow Cytometric Detection of Cell-Surface DR4 and DR5 Expressions

After treated with 5,7-dihydroxyflavone for 24 h, HepG2 cells were collected, washed twice with cold FACS (0.5% BSA in PBS) buffer, and incubated with mouse IgG1 anti-DR4 or anti-DR5 monoclonal antibodies for 60 min on ice. Subsequently, the cells were washed twice with FACS buffer and incubated with FITC-conjugated antimouse IgG antibody for 15 min in the dark on ice. After two further washes, the cells were analyzed by flow cytometry. A purified mouse IgG1 was used as isotype-matched control.

### 2.8. *In Vivo* Therapeutic Experiments

The animal study was performed according to the international rules considering animal experiments and the internationally accepted ethical principles for laboratory animal use and care. Balb/c female nude mice were inoculated subcutaneously with 4 × 10^6^ HepG2 cells in the right flank. When the average tumor volume reached about 150 mm^3^, mice were randomly divided into four groups of 9 animals in each group: Group1, vehicle control (0.5% sodium carboxymethyl cellulose, CMCNa) administered by oral gavage; Group 2, 5,7-dihydroxyflavone (30 mg/kg/d) administered by oral gavage; Group 3, TRAIL (10 mg/kg/d) administered i.p.; Group 4, 5,7-dihydroxyflavone + TRAIL, 5,7-dihydroxyflavone (30 mg/kg/d) administered by oral gavage and TRAIL (10 mg/kg/d) administered i.p.. For these experiments, 5,7-dihydroxyflavone was suspended in 0.5% CMCNa. Mice were treated for 28 days, and tumor volume was measured twice a week using vernier calipers. The tumor volume was calculated using the following formula: (long axis × short axis^2^)/2. On day 29, mice were killed, and tumors were removed and weighed. 

### 2.9. Statistical Analysis of Data

Three or more separate experiments were performed. Values were expressed as means ± standard deviations (SD). All statistical analysis was performed using the software SPSS 18.0 for Windows (Chicago, IL, USA). Statistical comparisons were made by one-way analysis of variance (ANOVA). *P*-values of <0.05 indicated statistical significance.

## 3. Results 

### 3.1. Combined 5,7-Dihydroxyflavone and TRAIL Treatment Induces Cytotoxicity in Cancer Cells but Not Normal Cells

**Figure 2:**
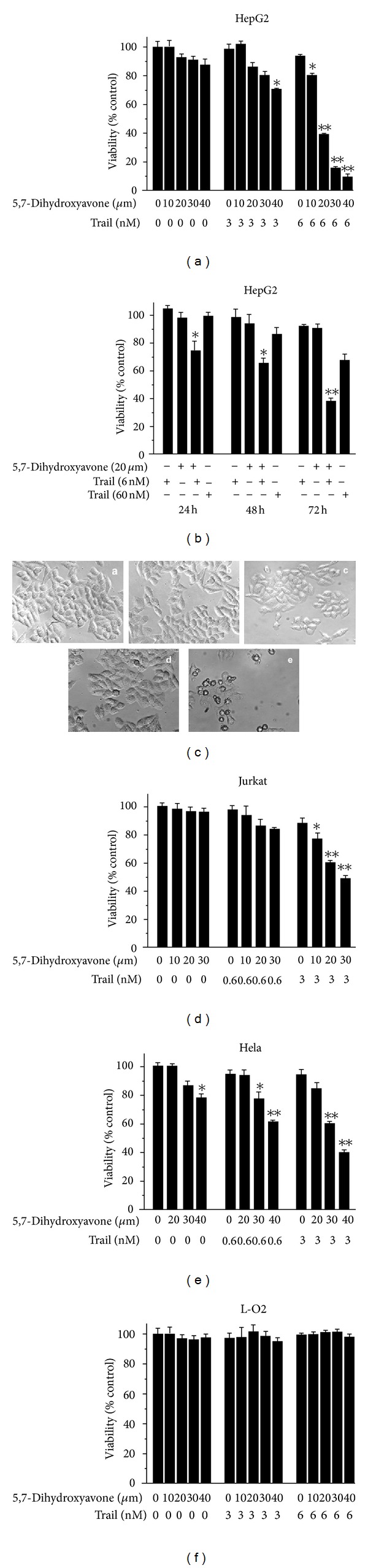
Interactive effects of 5,7-dihydroxyflavone and TRAIL on cell viability. (A), HepG2 cells were treated with various concentrations of 5,7-dihydroxyflavone and/or TRAIL. Cell viability was measured at the end of 72 h by MTT assay. Values marked with asterisk(s) are significantly different (*P < 0.05, **P < 0.01) from control. Data are mean ± SD. (B), HepG2 cells were treated with 5,7-dihydroxyflavone and/or TRAIL for indicated lengths of time. Cell viability was measured by MTT assay. Values marked with asterisk(s) are significantly different (*P < 0.05, **P < 0.01) from control. Data are mean ± SD. (C), light micrograph of HepG2 cells treated with 5,7-dihydroxyflavone and/or TRAIL for 24 h (×200). (a, control; b, 6 nM TRAIL; c, 20 *μ*M 5,7-dihydroxyflavone; d, 60 nM TRAIL; e, 6 nM TRAIL + 20 *μ*M 5,7-dihydroxyflavone). (D), Jurkat cells were treated with 5,7-dihydroxyflavone and/or TRAIL for 48 h and cell viability was measured by MTT assay. Values marked with asterisk(s) are significantly different (*P < 0.05, **P < 0.01) from control. Data are mean ± SD. (E), Hela cells were treated with 5,7-dihydroxyflavone and/or TRAIL for 72 h and cell viability was measured by MTT assay. Values marked with asterisk(s) are significantly different (*P < 0.05, **P < 0.01) from control. Data are mean ± SD. (F), L-O2 cells were treated with various concentrations of 5,7-dihydroxyflavone and/or TRAIL. Cell viability was measured by MTT assay. Data are mean ± SD.

To investigate the effects of 5,7-dihydroxyflavone on TRAIL-mediated cytotoxicity, we treated HepG2 cells with the indicated agents and subjected them to the MTT assay. As shown in Figure 2(a), treatment of HepG2 cells with 5,7-dihydroxyflavone or TRAIL alone for 72 h induced little cytotoxicity (~10%). Notably, simultaneous exposure of HepG2 cells to 5,7-dihydroxyflavone and TRAIL resulted in remarkably enhanced cytotoxicity. 

We also examined the cytotoxic effects of the combined treatment with TRAIL and 5,7-dihydroxyflavone for the indicated lengths of time. The results showed that a significant reduction of cell viability occurred in a time-dependent manner (Figure 2(b)). The cell viability of HepG2 cells treated with either TRAIL (6 nmol/L) or 5,7-dihydroxyflavone (20 *μ*mol/L) for 72 h was 93.61 ± 1.09% and 92.76 ± 2.36%, respectively, which decreased to 38.96 ± 1.25% with concurrent treatment. It is important to note that the combined treatment with 5,7-dihydroxyflavone (20 *μ*mol/L) and TRAIL (6 nmol/L) was more robust in inhibiting the cell viability than 60 nmol/L TRAIL.

The cell death was easily visualized by phase-contrast microscopy. As shown in Figure 2(c), the most conspicuous changes observed in cells with combined treatment included cell shrinkage and extensive detachment of cells from the cell culture substratum. These changes were absent in the cells treated with 5,7-dihydroxyflavone or TRAIL alone, even with 60 nmol/L TRAIL. 

In addition, we assessed whether synergism between 5,7-dihydroxyflavone and TRAIL occurs in other cancer cells, including human acute leukemia Jurkat T cells and human cervical carcinoma HeLa cells. The cells were treated with 5,7-dihydroxyflavone or TRAIL alone or combined for 72 h, and cell viability was analyzed using the MTT assay. As shown in Figures 2(d) and 2(e), 5,7-dihydroxyflavone augmented TRAIL-induced toxicity in both cell lines.

However, TRAIL combination treatment bears the risk of sensitizing otherwise TRAIL-resistant normal cells. We thus explored a potential cytotoxic effect of combined 5,7-dihydroxyflavone-TRAIL treatment in normal hepatocytes. 5,7-Dihydroxyflavone in conjunction with TRAIL did not impose any cytotoxicity on the nonmalignant cells (Figure 2(f)).

### 3.2. 5,7-Dihydroxyflavone Augments TRAIL-Induced Apoptosis in Tumor Cells

In order to confirm that the mode of cell death induced by 5,7-dihydroxyflavone-TRAIL treatment was indeed apoptosis, a number of biochemical and morphological markers of apoptosis were investigated. 

**Figure 3:**
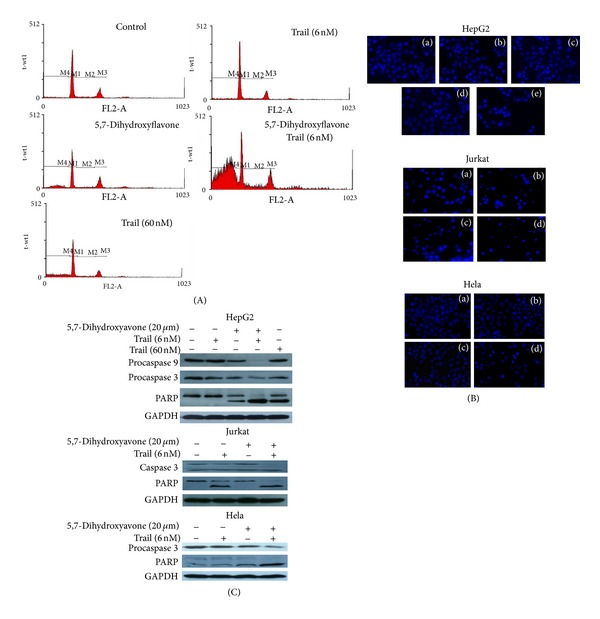
5,7-Dihydroxyflavone sensitized tumor cells to TRAIL-induced apoptosis. (A) HepG2 cells were treated with 5,7-dihydroxyflavone and/or TRAIL for 24 h. Apoptosis was assessed by propidium iodide DNA staining and flow cytometry. (B) HepG2, Jurkat or HeLa cells were treated with 5,7-dihydroxyflavone and/or TRAIL for 24 h. Apoptosis was assessed by hoechst33258 staining (×200) (HepG2 cells: a, control; b, 6 nM TRAIL; c, 20 *μ*M 5,7-dihydroxyflavone; d, 60 nM TRAIL; e, 6 nM TRAIL + 20 *μ*M 5,7-dihydroxyflavone; Jurkat and HeLa cells: a, control; b, 6 nM TRAIL; c, 20 *μ*M 5,7-dihydroxyflavone; d, 6 nM TRAIL + 20 *μ*M 5,7-dihydroxyflavone). (C) HepG2, Jurkat or HeLa cells were treated with 5,7-dihydroxyflavone and/or TRAIL for 24 h. Cell lysates were separated by SDS-PAGE, then procaspase-9, procaspase-3, and PARP proteins were detected by Western blot analysis. GADPH was used as loading control.

First, we analyzed the apoptotic effects by flow cytometric analysis to detect hypodiploid cell populations. As shown in Figure 3(A), the proportion of the sub-G1 peak was negligible in untreated HepG2 cells or those treated with 5,7-dihydroxyflavone (20 *μ*mol/L) or TRAIL (6 or 60 nmol/L) alone, whereas cotreatment of cells with 5,7-dihydroxyflavone (20 *μ*mol/L) and TRAIL (6 nmol/L) led to a markedly increased accumulation of sub-G1 phase cells (*P* < 0.001). Notably, the apoptotic rate of HepG2 cells treated with both 5,7-dihydroxyflavone and TRAIL was much higher than what would be expected if the effect was simply additive.

Next, we observed nuclei treated with 5,7-dihydroxyflavone and/or TRAIL using Hoechst33258 staining. As a single treatment, neither 5,7-dihydroxyflavone (20 *μ*mol/L) nor TRAIL (6 or 60 nmol/L) had any effect on the nuclei of HepG2 cells. The image of nuclei was similar to that of cells treated with the solvent DMSO. However, following combined treatment with 5,7-dihydroxyflavone (20 *μ*mol/L) and TRAIL (6 nmol/L) for 24 h, the appearance of condensed and fragmented nuclei in HepG2 cells was observed. Moreover, the increase in apoptosis in cotreated Jurkat and HeLa cell lines was also detected (Figure 3(B)). 

Caspases are known to act as important mediators of apoptosis and are also known to contribute to overall apoptotic morphology through the cleavage of various cell substrates [17]. We then investigate proforms of caspase-9 and caspase-3, and the subsequent proteolytic cleavage of PARP in HepG2 cells treated with 5,7-dihydroxyflavone (20 *μ*mol/L) and/or TRAIL (6 nmol/L) for 24 hours. As shown in Figure 3(C), Western blot analysis revealed that untreated HepG2 cells and those treated with 5,7-dihydroxyflavone (20 *μ*mol/L) or TRAIL (6 nmol/L) alone showed little or no decrease in procaspase-9 or procaspase-3. However, combined treatment with TRAIL and 5,7-dihydroxyflavone significantly decreased the procaspase levels. Interestingly, the combination of 5,7-dihydroxyflavone and TRAIL resulted in more processing of procaspase-9 and procaspase-3 than those treated with 60 nmol/L TRAIL. The almost complete cleavage of PARP, a downstream target of active caspase-3 which serves as a marker of apoptosis [18], in 5,7-dihydroxyflavone-TRAIL cotreated HepG2 cells coincided with the above results. In addition, cotreatment of Jurkt and HeLa cells with 5,7-dihydroxyflavone and TRAIL led to more processing of procaspase-3 and cleavage of PARP than treatment with either agent alone (Figure 3(C)). These results indicated that 5,7-dihydroxyflavone significantly enhanced TRAIL-induced apoptosis in tumor cell lines.

### 3.3. Effect of 5,7-Dihydroxyflavone on Expression of DR4 and DR5

**Figure 4:**
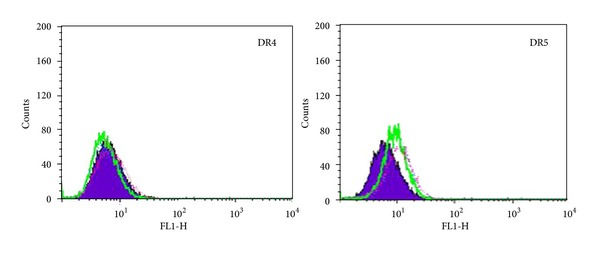
Influence of 5,7-dihydroxyflavone on DR4 and DR5 expressions. HepG2 cells were treated with or without 5,7-dihydroxyflavone for 24 h and then harvested for analysis of cell surface DR4 and DR5 by immunofluorescent staining and subsequent flow cytometry. Filled purple peaks, cells stained with a match isotype control; green line, untreated control; pink line, 20 *μ*mol/L 5,7-dihydroxyflavone; blue line, 40 *μ*mol/L 5,7-dihydroxyflavone.

TRAIL transmits the proapoptotic signal by interacting with DR4 and DR5 [4]. Because TRAIL-induced apoptosis in HepG2 cells was enhanced by 5,7-dihydroxyflavone, we considered the possibility that 5,7-dihydroxyflavone might augment TRAIL-induced apoptosis by modulating the expression of TRAIL death receptors. We examined HepG2 cells for the expression of DR4 and DR5 and the effect 5,7-dihydroxyflavone has on their expression by flow cytometry. However, treatment with 5,7-dihydroxyflavone for 24 hours did not increase cell-surface expression of proapoptotic TRAIL receptors (Figure 4). 

### 3.4. Effect of 5,7-Dihydroxyflavone on Expression of c-FLIP

**Figure 5:**
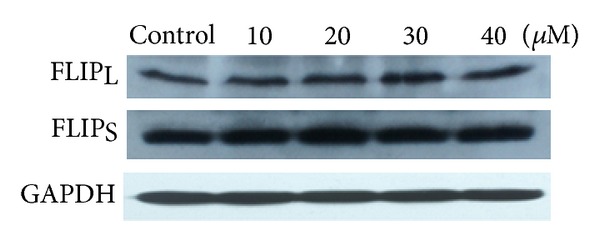
Influence of 5,7-dihydroxyflavone on the expression of c-FLIP. HepG2 cells were treated with the indicated concentrations of 5,7-dihydroxyflavone for 24 h. Western blotting was performed to determine the protein levels of c-FLIP. GADPH was used as loading control.

Overexpression of cellular FADD-like interleukin-1b-converting enzyme inhibitory protein (c-FLIP) can confer resistance to TRAIL [19]. c-FLIP, structurally similar to caspase-8, can be recruited into the DISC by competing with caspase-8, resulting in inhibition of caspase-8 activation and inhibition of subsequent apoptosis [20]. The expression level of c-FLIP, therefore, may determine the sensitivity of cancer cells to TRAIL-induced apoptosis [21]. We then examined whether 5,7-dihydroxyflavone affects the expression of c-FLIP. The results showed that the protein levels of both c-FLIP_L_ and FLIP_S_, the major splice forms of c-FLIP [20], were unchanged after 5,7-dihydroxyflavone treatment (Figure 5). 

### 3.5. Regulation of Bcl-2 Family Members by 5,7-Dihydroxyflavone

**Figure 6:**
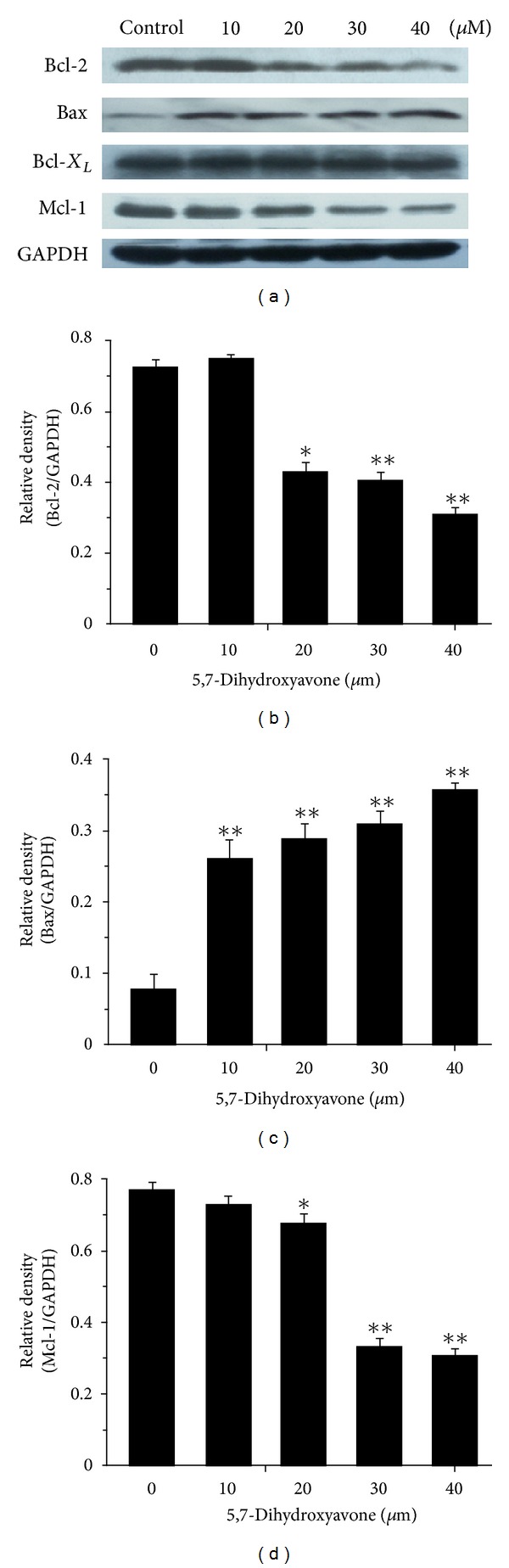
Influence of 5,7-dihydroxyflavone on the expression of Bcl-2, Bax, Bcl-X_*L*_, and Mcl-1. (a) HepG2 cells were treated with the indicated concentrations of 5,7-dihydroxyflavone for 24 h. Western blotting was performed to determine the protein levels of Bcl-2, Bax, Bcl-X_*L*_, and Mcl-1. GADPH was used as loading control. (b)–(d) the relative densities of these proteins were determined following densitometric measurements of the specific protein bands and normalization against the value of GAPDH. The asterisk indicates a significant difference (*P < 0.05, **P < 0.01) compared with control. Data are mean ± SD.

The Bcl-2 protein family has been demonstrated to play a critical role in the regulation of apoptosis [22]. The ratio of Bax/Bcl-2 especially is a decisive factor and plays an important role in determining whether cells will undergo death or survival [23]. We, therefore, examined the effects of 5,7-dihydroxyflavone on the expression levels of Bcl-2 family members. The Western blot analysis showed that 5,7-dihydroxyflavone downregulated the expression of antiapoptotic proteins Bcl-2 and Mcl-1 in HepG2 cells in a concentration-dependent manner. In contrast, the protein level of Bax was upregulated by treatment with 5,7-dihydroxyflavone (Figure 6). 

### 3.6. Inhibition of IAPs by 5,7-Dihydroxyflavone

**Figure 7:**
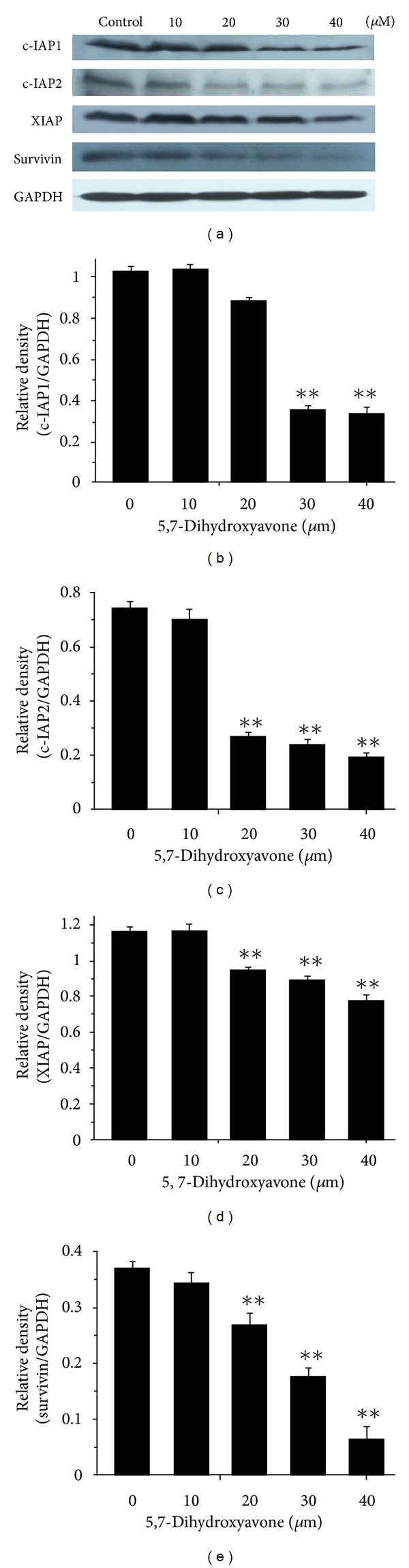
Influence of 5,7-dihydroxyflavone on the expression of c-IAP1, c-IAP2, XIAP, and Survivin. (a) HepG2 cells were treated with the indicated concentrations of 5,7-dihydroxyflavone for 24 h. Western blotting was performed to determine the protein levels of c-IAP1, c-IAP2, XIAP, and Survivin. GADPH was used as loading control. (b)–(e) the relative densities of these proteins were determined following densitometric measurements of the specific protein bands and normalization against the value of GAPDH. The asterisk indicates a significant difference (**P < 0.01) compared with control. Data are mean ± SD.

IAPs play a major role in inhibiting caspase activation and apoptosis [24]. Since 5,7-dihydroxyflavone enhanced TRAIL-induced apoptosis by activation of caspases, we sought to examine the effects of 5,7-dihydroxyflavone on the expression of IAPs in HepG2 cells. We found that the protein levels of all the tested IAPs, including c-IAP1, c-IAP2, XIAP, and Survivin, were significantly decreased in response to treatment with 5,7-dihydroxyflavone. These data suggested that inhibition of IAPs by 5,7-dihydroxyflavone may be one of the mechanisms regulating sensitivity of HepG2 cells to TRAIL (Figure 7). 

### 3.7. Effect of 5,7-Dihydroxyflavone on Phosphorylation of Akt, STAT3, and MAPK

**Figure 8:**
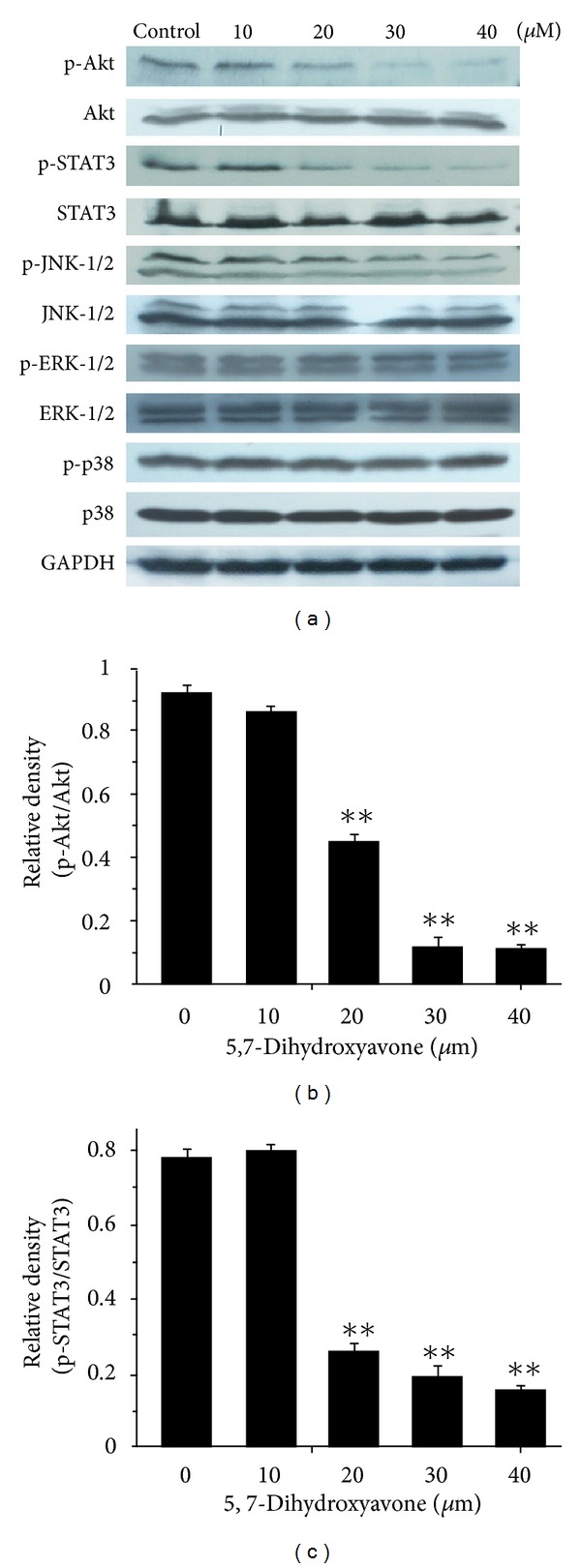
Influence of 5,7-dihydroxyflavone on the activation of Akt, STAT3, and MAPK. (a) HepG2 cells were treated with the indicated concentrations of 5,7-dihydroxyflavone for 24 h. Western blotting was performed to determine the phosphorylation levels of Akt, STAT3, and mitogen-activated protein kinase (MAPK). GADPH was used as loading control. (b, c), the relative densities of phospho-Akt and phosphor-STAT3 were determined following densitometric measurements of the specific protein bands and normalization against the value of total Akt and STAT3, respectively. The asterisk indicates a significant difference (**P < 0.01) compared with control. Data are mean ± SD.

In order to further analyze modulators of TRAIL sensitivity in HepG2 cells, we perform Western blotting to determine the phosphorylation levels of Akt, STAT3, and mitogen-activated protein kinase (MAPK) in HepG2 cells treated with 5,7-dihydroxyflavone. As shown in Figure 8, constitutive phosphorylation of Akt and STAT3 was observed in untreated cells, which decreased after 5,7-dihydroxyflavone treatment for 24 hours. Constitutive phosphorylation of JNK1/2/3, ERK, and p38 was also observed before treatment; however, the activation of JNK1/2/3 was only slightly reduced and the activation of ERK1/2 and p38 was not altered. Its possible that phospho-Akt and phospho-STAT3 contribute to TRAIL resistance in HepG2 cells, the effects of which were reduced by 5,7-dihydroxyflavone treatment. 

### 3.8. Combination of 5,7-Dihydroxyflavone and TRAIL Inhibits HepG2 Xenograft Tumor Growth* In Vivo*

**Figure 9:**
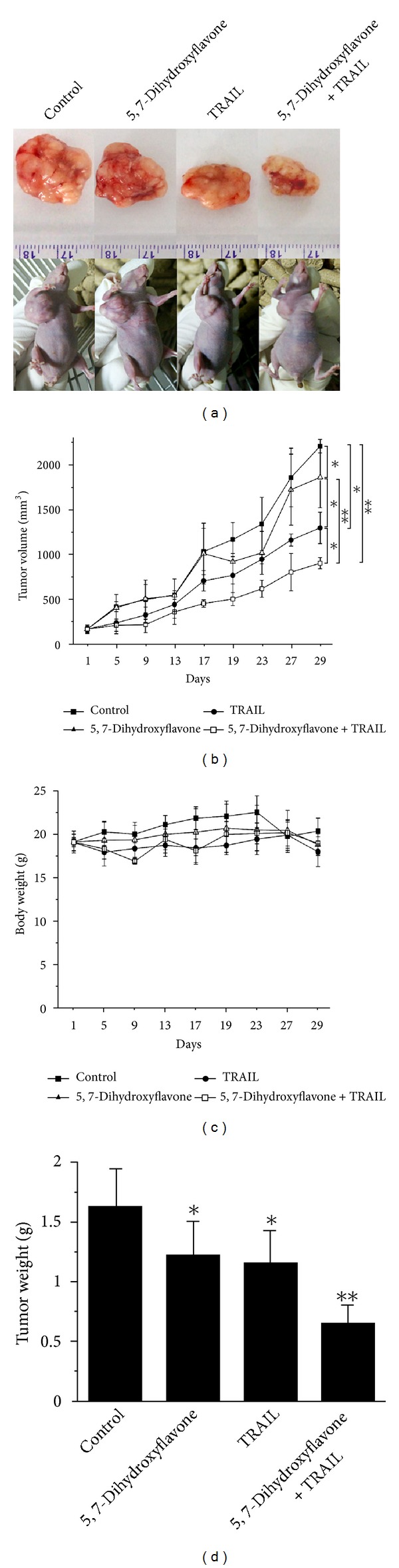
5,7-Dihydroxyflavone synergized with TRAIL in the treatment of hepatocarcinoma xenografts. HepG2 cells were implanted into athymic mice subcutaneously. When the average tumor volume reached about 150 mm^3^, mice (*N* = 9) were treated for 28 days with vehicle control, 5,7-dihydroxyflavone (30 mg/kg/d) by oral gavage, TRAIL (10 mg/kg/d) i.p., and 5,7-dihydroxyflavone (30 mg/kg/d) by oral gavage + TRAIL(10 mg/kg/d) i.p.. (a) and (d) on day 29, tumors were isolated and weighed. (b) the tumor growth was monitored twice a week. (c) animal weights were recorded during the 28-day treatment. The asterisk indicates a significant difference (*P < 0.05, **P < 0.01) compared with control. Data are mean ± SD.

To determine whether 5,7-dihydroxyflavone in combination with TRAIL could inhibit tumor growth *in vivo*, HepG2 tumor-bearing mice were treated for 28 days with 5,7-dihydroxyflavone (30 mg/kg/d), TRAIL (10 mg/kg/d), the combination of 5,7-dihydroxyflavone and TRAIL, or vehicle control. We found that 5,7-dihydroxyflavone inhibited HepG2 tumor growth and strengthened HepG2 tumor growth inhibition induced by TRAIL (*P* < 0.05) (Figures 9(a), 9(b), and 9(d)), demonstrating an enhanced inhibitory effect of 5,7-dihydroxyflavone/TRAIL combination treatment on the *in vivo* model of hepatocarcinoma. The body weight of mice in each group did not show any significant difference over the 28-day experiment (Figure 9(c)), suggesting no apparent toxicity due to 5,7-dihydroxyflavone and TRAIL treatment.

## 4. Discussion 

TRAIL, a novel member of the TNF superfamily, has recently drawn considerable interest as a potential effective anticancer therapeutic agent because it shows selective toxicity to a wide range of malignant tumor cells with minimal toxicity to normal cells [25]. Unfortunately, considerable numbers of cancer cells, especially some highly malignant tumors, are resistant to apoptosis induction by TRAIL [26], and some cancer cells that were originally sensitive to TRAIL-induced apoptosis can become resistant after repeated exposure [27]; even some of the TRAIL-resistant cells express the TRAIL death receptors [28]. The cytotoxic activity of TRAIL alone, therefore, may be insufficient for cancer therapy. Thus, researchers are seeking to identify effective sensitizers for TRAIL-induced apoptosis that may allow cancer cells to recover TRAIL sensitivity [8]. 

Natural products have played a highly significant role over the years in the discovery of new drugs [29, 30]. This is particularly evident in the treatment of cancers and infectious diseases in which more than 60% and 75% of drugs, respectively, are of natural origin [31]. 5,7-Dihydroxyflavone has demonstrated antiproliferative activity in human cancer cell lines [32–34]. 

Our present study revealed that 5,7-dihydroxyflavone is a potent sensitizer to TRAIL and the combination of 5,7-dihydroxyflavone and TRAIL is selectively active in cancer cells without affecting normal hepatocytes, indicating that the combination of 5,7-dihydroxyflavone and TRAIL may be an effective approach for the treatment of cancer. It is important to note that the combined treatment with 5,7-dihydroxyflavone (20 *μ*mol/L) and TRAIL (6 nmol/L) was more robust in inducing apoptosis of HepG2 cells than 60 nmol/L TRAIL. The results mean that the combination of these agents generates an effect that is more than simply additive. 

It has been reported that the mechanisms of TRAIL resistance may include low-level expression of TRAIL death receptors [3, 6], high expression of antiapoptotic proteins (cFLIP, Bcl-2 family and IAPs) [35, 36], and activation of antiapoptotic signaling (Akt, MAPK, et al.) [37–39]. 

In the search of the molecular mechanisms involved in the sensitization of 5,7-dihydroxyflavone, we first examined the cell surface expression levels of DR4 and DR5. The results showed that treatment with 5,7-dihydroxyflavone for 24 hours did not alter the expression of DR4 or DR5 on HepG2 cells. It has been reported that posttranslational modification and localization of DR4 and DR5 may also have an important role in determining TRAIL sensitivity [4, 40, 41]. Therefore, additional assays are needed to explore whether 5,7-dihydroxyflavone sensitized HepG2 cells to TRAIL via altering the posttranslational modification and localization of TRAIL death receptors. 

We then went on to screen the changes in apoptosis regulatory proteins. In our study, 5,7-dihydroxyflavone has no effect on the expression of c-FLIP. However, one important finding from our study is that treatment with 5,7-dihydroxyflavone leads to downregulation of Bcl-2, Mcl-1, and IAPs (c-IAP1, c-IAP2, XIAP, and Survivin) and upregulation of Bax. The antiapoptotic members of the Bcl-2 family like Bcl-2, Bcl-X_*L*_, and Mcl-1 are associated with the mitochondrial outer membrane and stabilize mitochondrial integrity [22]. Overexpression of these proteins inhibits the activation of the mitochondrial pathway and subsequently renders tumor cells refractory to TRAIL-induced apoptosis [42, 43]. Bcl-2 has been shown to form a heterodimer complex with the proapoptotic member Bax, thereby neutralizing its proapoptotic effects. Therefore, the ratio of Bax:Bcl-2 is a decisive factor and plays an important role in determining whether cells will undergo death or survival [23, 44]. In the present study, 5,7-dihydroxyflavone increased Bax/Bcl-2 ratio in HepG2 cells. The IAPs are an important family of apoptosis regulating proteins, with Survivin and XIAP as prominent members [45]. IAPs have been shown to block both the mitochondrial and death-receptor-mediated pathways of apoptosis by directly binding to and inhibiting both the initiator and effector caspases [24]. We found that the protein levels of all the tested IAPs significantly decreased in response to treatment with 5,7-dihydroxyflavone. 

Several other factors may modulate the death signal of the TRAIL pathway. Akt, also known as PKB, plays a major role in regulation of cell growth, apoptosis, and survival [46]. It has been shown that elevated Akt activity renders TRAIL-sensitive cells to be TRAIL resistant and that IAPs are regulated by Akt [47]. In the present study, HepG2 cells expressed constitutively active Akt, which were inhibited by 5,7-dihydroxyflavone. Meanwhile, the downregulation of phospho-STAT3 was observed after 5,7-dihydroxyflavone treatment in a concentration-dependent manner. This finding is consistent with the reports that abrogation of constitutive activation of STAT3 by AG490 sensitizes human hepatoma cells to TRAIL-induced apoptosis [48]. The MAP kinases are a superfamily of proteins that transmit signaling cascades from extracellular stimuli into cells, including three family members: the extracellular signal-regulated kinase (ERK), c-jun N-terminal protein kinases (JNK), and the p38-MAPK [49]. When we tested for changes in members of the MAPK signaling pathways following treatment with 5,7-dihydroxyflavone, the activation of JNK was only slightly reduced, and activation of p38 and ERK was not significantly changed. In view of these findings, it is likely that 5,7-dihydroxyflavone promotes TRAIL-induced apoptosis independent of MAPK pathway. 

Furthermore, we used a hepatocarcinoma xenograft model to investigate whether 5,7-dihydroxyflavone could acte synergistically with TRAIL to reduce tumor burden *in vivo*. Our data showed apparent synergy of 5,7-dihydroxyflavone and TRAIL with respect to suppression of tumor growth in mice. At doses resulting in significant suppression of tumor xenograft growth, the combination of 5,7-dihydroxyflavone and TRAIL was well tolerated in mice. This paper provides the first *in vivo* proof of concept data demonstrating the efficacy of the combination of 5,7-dihydroxyflavone and TRAIL to reduce tumor burden in mice.

In conclusion, our results provide evidence that the combined treatment with 5,7-dihydroxyflavone and TRAIL at subtoxic concentrations induced strong apoptotic response in human hepatocarcinoma HepG2 cells, acute leukemia Jurkat T cells, and cervical carcinoma HeLa cells, but did not affect the viability of normal hepatocytes. 5,7-dihydroxyflavone effectively recovers TRAIL sensitivity in human hepatocarcinoma HepG2 cells via multiple modulators, including Bcl-2, Mcl-1, IAPs, Akt, and STAT3. Moreover, 5,7-Dihydroxyflavone and TRAIL were well tolerated in mice and the combination of 5,7-Dihydroxyflavone and TRAIL reduced tumor burden in a hepatocarcinoma xenograft model. Therefore, in terms of a clinical perspective, the combination of 5,7-dihydroxyflavone with TRAIL may be a novel strategy for the treatment of a variety of human cancers that warrants further investigation.
